# Genomic Analysis of Companion Rabbit *Staphylococcus aureus*

**DOI:** 10.1371/journal.pone.0151458

**Published:** 2016-03-10

**Authors:** Mark A. Holmes, Ewan M. Harrison, Elizabeth A. Fisher, Elizabeth M. Graham, Julian Parkhill, Geoffrey Foster, Gavin K. Paterson

**Affiliations:** 1 Department of Veterinary Medicine, University of Cambridge, Cambridge, United Kingdom; 2 Department of Medicine, University of Cambridge, Cambridge, United Kingdom; 3 School of Biological, Biomedical and Environmental Sciences, University of Hull, Kingston upon Hull, United Kingdom; 4 School of Veterinary Medicine, University of Glasgow, Glasgow, United Kingdom; 5 The Wellcome Trust Sanger Institute, Wellcome Trust, Genome Campus, Hinxton, United Kingdom; 6 Scottish Agricultural College Consulting Veterinary Services, Inverness, United Kingdom; Institut National de la Recherche Agronomique, FRANCE

## Abstract

In addition to being an important human pathogen, *Staphylococcus aureus* is able to cause a variety of infections in numerous other host species. While the *S*. *aureus* strains causing infection in several of these hosts have been well characterised, this is not the case for companion rabbits (*Oryctolagus cuniculus*), where little data are available on *S*. *aureus* strains from this host. To address this deficiency we have performed antimicrobial susceptibility testing and genome sequencing on a collection of *S*. *aureus* isolates from companion rabbits. The findings show a diverse *S*. *aureus* population is able to cause infection in this host, and while antimicrobial resistance was uncommon, the isolates possess a range of known and putative virulence factors consistent with a diverse clinical presentation in companion rabbits including severe abscesses. We additionally show that companion rabbit isolates carry polymorphisms within *dltB* as described as underlying host-adaption of *S*. *aureus* to farmed rabbits. The availability of *S*. *aureus* genome sequences from companion rabbits provides an important aid to understanding the pathogenesis of disease in this host and in the clinical management and surveillance of these infections.

## Introduction

*Staphylococcus aureus* is a major human and veterinary pathogen, responsible for a wide range of diseases. Among economically important livestock animals these diseases include mastitis in dairy cows and small ruminants, lameness in commercial broiler chickens and virulent epidemics of skin abscesses, mastitis and septicaemia in farmed rabbits (*Oryctolagus cuniculus*). *S*. *aureus* colonisation and infection of animals is not only important from the perspective of animal wellbeing and economic impact but may also lead to zoonotic infection of humans [1, 2]. Although also recognised as a versatile and virulent pathogen among companion rabbits, in particular as a cause of severe abscesses [3], few data are available on the *S*. *aureus* strains causing disease in this host. This is despite the popularity of rabbits as a companion animal; for instance the population of companion rabbits in the UK is estimated at ~ 1 million [4]. Furthermore *S*. *aureus*, including methicillin-resistant *S*. *aureus* (MRSA) is a well-documented pathogen among other companion animals such as cats, dogs and horses, [5–7]. Interest in the *S*. *aureus* population among companion rabbits is further heightened by the discovery of emergent *mecC* MRSA in a farmed rabbit [8], and sporadic reports of MRSA in companion rabbits [9], including livestock-associated clonal complex 398 MRSA [10] and Panton-Valentine Leucocidin-positive isolates [11]. Furthermore, rabbits are a frequently used experimental model for *S*. *aureus* infections and a better understanding of the natural bacterial-host interactions in this setting may facilitate improved model systems.

In order to address this paucity of data on the *S*. *aureus* population among companion rabbits we have genome sequenced a collection of companion and research unit *S*. *aureus* from this host species in the United Kingdom. This novel genome-level study provides insight into host-pathogen interactions, antimicrobial resistance and the phylogenetics of *S*. *aureus* among rabbits. These data will inform clinical management in rabbits and the future surveillance of this widespread and important pathogen.

## Materials and Methods

### Bacterial isolates and antimicrobial susceptibility

A request for *S*. *aureus* isolates from companion rabbits was made to personal contacts and veterinary diagnostic laboratories in the UK. Isolates where collected by veterinary microbiology laboratories in the course of their routine diagnostic work, with the study approved by the Department of Veterinary Medicine, University of Cambridge Ethics and Welfare Committee (reference: CR76 Collection of *S*. *aureus* isolates from domestic and wild animals for genome sequencing). The resultant ten isolates that were collected and their associated details are shown in Table 1. Antimicrobial susceptibility testing was performed using the Staph AST-P620 card on the Vitek 2 system (bioMérieux, Basingstoke, UK) following the manufacturer’s instructions with *S*. *aureus* NCTC6571 and NCTC12493 as control strains.

**Table 1:** Rabbit isolates included in this study.

Isolate name	ERA Accession	Coverage	Biosample	Assembly Accessions	Geographical location	Site of isolation	Date of isolation	ST^1^	CC^2^	*spa* type^3^	Phenotypic resistance^4^	Resistance genes/mutations	Additional notes
FP01	ERR387096	163x	SAMEA1929514	FJNS01000001-FJNS01000044	Manchester area, England	Not known	Apr-2013	30	30	t021	benzylpenicillin	*blaZ*, *tet(38)*, *norA*	same animal as FP02
FP02	ERR387097	156x	SAMEA1929515	FJNW01000001-FJNW01000043	Manchester area, England	Not known	Apr-2013	30	30	t021	benzylpenicillin	*blaZ*, *tet(38)*, *norA*	same animal as FP01
557472	ERR387166	113x	SAMEA1929647	FJNT01000001-FJNT01000024	England	Ventral vulva abscess	Jun-2013	**3126**	291	t1614	benzylpenicillin	*blaZ*, *tet(38)*, *norA*	
M1970/98/1	ERR387195	160x	SAMEA1929516	FJNU01000001-FJNU01000024	Scotland	Lesion	1998	**3120**	425	t13114	susceptible	*tet(38)*, *norA*	
M503044/99/1	ERR387196	147x	SAMEA1929517	FJNP01000001-FJNP01000029	Scotland	Sub-cutaneous abscess	1999	121	121	t645	susceptible	*tet(38)*, *norA*	
543471	ERR387256	90x	SAMEA1929646	FJNQ01000001-FJNQ01000011	England	Darcocystitis	Feb-2013	6	6	t5413	benzylpenicillin, fusidic acid	*blaZ*, *tet(38)*, *norA;* H457Y in elongation factor G	
559622	ERR387257	101x	SAMEA1929648	FJNN01000001-FJNN01000024	England	Skin infection	Jun-2013	15	15	t2574	benzylpenicillin	*blaZ*, *tet(38)*, *norA*	
61908	ERR494744	134x	SAMEA2298602	FJNO01000001-FJNO01000014	Stirlingshire, Scotland	Nasal sample at post-mortem	Sep-2009	**3092**	425	**t15410**	susceptible	*tet(38)*, *norA*	feral rabbit
68850	ERR494745	144x	SAMEA2298603	FJNV01000001-FJNV01000032	Glasgow, Scotland	Chest cavity abscess	Jun-2012	39	30	**t15409**	benzylpenicillin	*blaZ*, *tet(38)*, *norA*	experimental research unit
68901	ERR494746	130x	SAMEA2298604	FJNR01000001-FJNR01000031	Glasgow, Scotland	Lower jaw abscess	Jun-2012	2257	22	t1977	benzylpenicillin	*blaZ*, *tet(38)*, *norA*	experimental research unit

^1^ Multi-locus sequenced type (new multi-locus sequence types shown in bold)

^2^ MLST clonal complex assigned by e-Burst

^3^ New *spa* types shown in bold

^4^ Tested against: benzylpenicillin, cefoxitin, oxacillin, ciprofloxacin, erythromycin, chloramphenicol, daptomycin, fusidic acid, gentamicin, linezolid, mupirocin, nitrofurantoin, rifampicin, teicoplanin, tetracycline, tigecycline, trimethoprim, vancomycin and clindamycin as well as inducible resistance to clindamycin.

### Genome sequencing and analysis

Genomic DNA was extracted using the MasterPure™ Gram Positive DNA Purification Kit (Cambio, Dry Drayton, UK) from overnight cultures grown from single colonies in 5 ml of tryptic soy broth overnight at 37°C. Illumina library preparation was carried out as described previously [12], and genome sequencing using Hi-Seq 2000 performed following the manufacturer’s standard protocols (Illumina, Little Chesterfield, UK). Nucleotide sequences been deposited in the European Nucleotide Archive, accession numbers provided in Table 1. Genome assembly was performed *de novo* using Velvet [13] and antimicrobial resistance genes and virulence factors identified using BLAST and ResFinder [14]. Genome-derived multi-locus sequence types (MLST) were assigned as described previously [15]. The phylogenetic relationships among the isolates was assessed using core genome (cg)MLST using SeqSphere^+^ software (Ridom GmbH, Münster Germany) as described previously [16] and including twenty-eight reference genomes to place the rabbit isolates within the context of the wider *S*. *aureus* population. 1475 core genome loci found in all isolates were used. *spa* typing was performed using Sanger sequencing of PCR products using primers spa-1113f (5'- TAA AGA CGA TCC TTC GGT GAG C -3') and spa-1514r (5'- CAG CAG TAG TGC CGT TTG CTT -3') as per Ridom GmbH (Würzburg, Germany).

## Results

### Study strains, multi-locus sequence types and *spa* types

Ten rabbit *S*. *aureus* isolates collected between 1998 and 2013 in the UK were included in this study, Table 1. Eight were from companion rabbits including two isolates from the same rabbit, with a further two isolates from research unit rabbits. MLST showed the ten isolates belonged to nine different sequence types, the only duplication of ST being the two isolates from the same rabbit which both belonged to ST30. Three new ST were identified in this study; ST3092 and ST3120 being single locus variants (SLV) of ST425 in *aroE* and *tpi* respectively, whilst ST3126 is a SLV of ST291 in *tpi*. These STs belonged to eight clonal complexes, Table 1. Similarly to MLST, nine different *spa* types were found among the ten isolates, the only duplication again being the two isolates from the same rabbit. Two new *spa* types, t15409 and t15410 were found.

### Whole genome phylogenetic analysis

**Fig 1:**
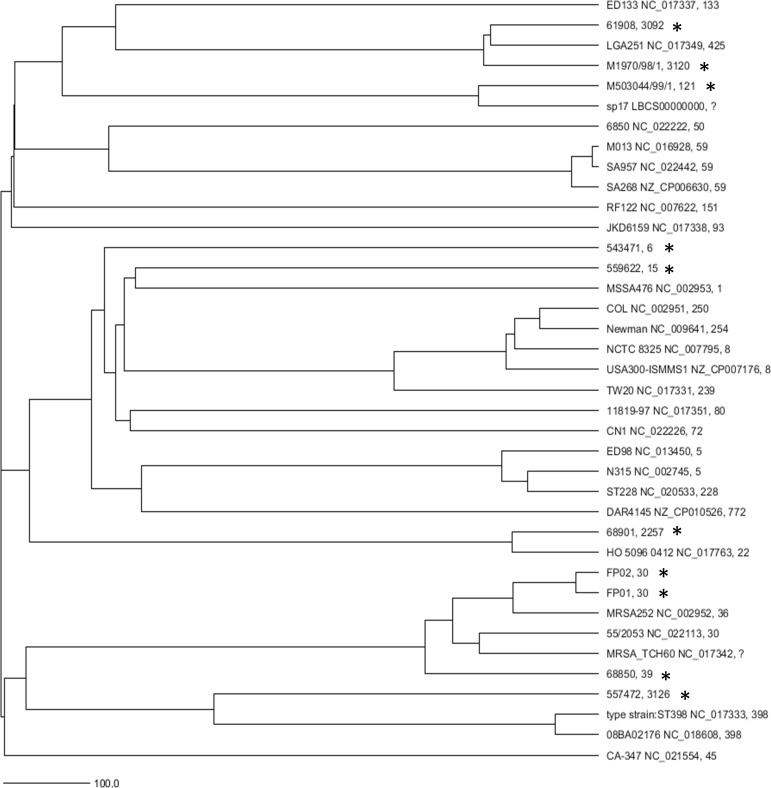
Clonal relationships among rabbit *S*. *aureus* and their context within the wider *S*. *aureus* population. A phylogenetic dendrogram (UPGMA) generated from the allelic profiles of 1475 cgMLST target genes, based on (16) and comprising the ten rabbit isolates from this study and twenty-eight reference *S*. *aureus* genomes from Genbank. Rabbit isolates are denoted by *, isolate name and Genbank accession provided for the reference genomes. The last figure in the text line indicates the multi-locus sequence type of each isolate, where available. The scale bar indicates the number of differing alleles comprising the calculated distance.

In agreement with the diversity indicated by MLST, whole genome analysis using cgMLST across 1475 loci showed a diverse population among rabbit isolates, Fig 1. The average pairwise difference in allele profile between rabbit isolates was 1271 alleles, representing 86% of the core genome loci assessed. The two isolates from the same rabbit, FP01 and FP02 differed in 51 alleles and the largest pairwise difference in profile was 1402 (95% of the 475 core loci analysed). Inclusion in the analysis of twenty-eight reference *S*. *aureus* genomes showed the rabbit isolates to be distributed across the wider *S*. *aureus* population, Fig 1

### Antimicrobial Resistance and resistance determinates

Resistance to benzylpenicillin occurred in seven of the isolates and correlated with the presence of *blaZ*, Table 1. Resistance to other antimicrobials was restricted to a single isolate, 543471, showing fusidic acid resistance, Table 1. This resistance correlated with a single amino acid substitution, H457Y in elongation factor G. Whilst no other phenotypic resistance was seen, all ten isolates were positive for the efflux pump genes *tet*(38) and *norA*.

### Virulence factors and markers of host adaption

The sequenced rabbit isolates were assessed for the presence or absence of *S*. *aureus* virulence factors, Table 2. Several genes, including those encoding α-(*hla*), β-(*hlb*) and γ-haemolysins (*hlgACB*) were present in all the isolates with others present in a subset, Table 2. None of the ten isolates possessed the genes encoding for Panton-Valentine Leucocidin but isolates FP01 and FP02 were both positive for toxic shock syndrome toxin-1. Four of the strains were positive for the phage-encoded immune evasion genes, *sak* and *scn* which are taken as indicative of strains of human origin. Among clinical rabbit isolates from commercial rabbitries in mainland Europe, Viana *et al*. have demonstrated a critical role in host adaption for polymorphisms in *dltB*, encoding the d-alanine teichoic acid esterification protein [17]. We therefore compared the DltB sequence in our isolates to that from human isolated *S*. *aureus*. Every rabbit isolate in our collection had at least one amino acid polymorphism in DltB, Table 2. These comprised both novel and previously described polymorphisms including the experimentally validated T113K *dltB* mutation [17].

**Table 2:** Distribution of virulence factors and *dltB* mutations among rabbit *S*. *aureus*.

	*fnbA*	*fnbB*	*sdrD*	*sdrE*	*efb*	*cna*	*sea*	*seb*	*sec*	*seg*	*sei*	*sen*	*seo*	*sep*	*pvlFS*	*tsst*	*sak*	*scn*	*dltB* polymorphisms^1^
FP01	+	-	-	-	+	+	+	+	-	-	+	+	+	+	-	+	+	+	**I227V** Y346H
FP02	+	-	-	-	+	+	+	+	-	-	+	+	+	+	-	+	+	+	**S48F** Y346H
557472	-	+	-	-	+	-	+	-	-	-	-	-	-	+	-	-	+	+	*405Q
M1970/98/1	+	+	+	+	-	+	-	-	-	-	-	-	-	+	-	-	-	-	**G5S** Q231R *405Q
M503044/99/1	-	+	+	-	-	+	+	-	+	-	+	+	-	-	-	-	-	-	T113K Y250H *405Y
543471	+	+	-	+	-	+	+	-	-	-	-	-	-	+	-	-	+	+	Y346H
559622	+	+	+	+	+	-	-	-	-	-	-	-	-	-	-	-	-	-	**L127S F221L** Y346C
61908	+	-	-	+	-	+	-	-	-	-	-	-	-	+	-	-	-	-	T113K *405Q
68850	+	-	-	-	+	+	-	-	-	-	-		+	+	-	-	-	-	**V328M** Y346H
68901	+	+	+	+	+	+	-	-	-	+	-	+	+	+	-	-	-	-	Y346C

^1^ as ascribed by comparison to MRSA252 *dltB* (locus tag SAR_RS04555 Accession NC_002952 REGION: 931760.932974). New mutations, not described previously by Viana *et al*. (2015) shown in bold.

Further genes present in all ten isolates but not displayed in table: *coa*, *nuc*, *spa*, *clfA*, *clfB*, *icaRABC*, *hla*, *hlb*, *hlgACB*, *eta*, *isb*, *sdrH*, *ebh*, *fib*, *ebpS*, *sdrC*

## Discussion

To gain insight into the molecular epidemiology and disease pathogenesis of rabbit staphylococcosis we have genome sequenced a collection of *S*. *aureus* isolates from companion rabbits. Strain typing by MLST and *spa* typing showed a diverse population of isolates with no duplication of ST or *spa* type between isolates from different animals and minimal overlap even at the level of MLST clonal complex. This finding of a diverse *S*. *aureus* population able to infect rabbits was further supported by the use of high-resolution whole genome analysis using cgMLST. Bacterial diversity extended to two isolates from the same individual rabbit which differed by 51 alleles and demonstrates that the within host diversity of *S*. *aureus* described previously in humans and dogs [18–21] extends to the rabbit host also. This diversity among the isolates indicates that a variety of *S*. *aureus* lineages are able to cause disease in companion rabbits, with no strong suggestion that any lineages are predominant, albeit based on a relatively small sample. However, the finding that this relatively small collection led to the identification of three novel STs and two novel *spa* types strongly indicates that the *S*. *aureus* population among this host has been poorly sampled to date and includes strains rare in humans and other animals.

With the exception of penicillin resistance, present in the majority of isolates and which correlated with the possession of *blaZ*, antimicrobial resistance was scarce. No MRSA isolates were found and the only additional phenotypic resistance was a single isolate resistant to fusidic acid. This isolate possessed a single amino acid substitution in elongation factor G which has previously been found associated with fusidic acid resistance in naturally occurring clinical isolates and in experimentally selected resistance mutants [22–25]. Furthermore, when introduced into a susceptible strain on a plasmid, this mutant *fusA* allele confers fusidic acid resistance [22]. All ten strains were positive for *norA*, a multidrug efflux pump which confers resistance to ciprofloxacin among a broad spectrum of agents [26] and *tet*(38), an efflux pump conferring tetracycline resistance [27]. In both cases however, phenotypic resistance is associated with mutations leading to over-expression which likely explains the absence of phenotypic resistance in these rabbit isolates [26, 27]. The presence of these genes in rabbit isolates, however, indicates the potential for such resistance to manifest in the future.

The importance of *dltB* polymorphisms in host adaption of *S*. *aureus* to rabbits has been demonstrated previously with a single amino acid substitution (T113K) sufficient to confer virulence in rabbits to a human ST121 isolate otherwise avirulent in that host [17]. Furthermore, while *dltB* is highly conserved in human isolates, thirty-nine rabbit isolates belonging to a range of STs and CCs all contained one or more non-synonymous SNPs in *dltB* thus suggesting convergent evolution among rabbit-adapted *S*. *aureus* [17]. All ten rabbit isolates in our collection carried at least one amino acid polymorphism in DltB with each strain encoding a different pattern of polymorphism(s) to each other. Two isolates belonging to ST121 and ST15 carried the experimentally validated T113K substitution. A second distinct *dltB* allele containing two SNPs and associated with the *S*. *aureus* ST96 rabbit clone was also shown experimentally to confer virulence in rabbits [17]. While one of those two SNPs, *405Q, was present in some of the isolates reported here, the second SNP, K402R, was not. We show therefore that host-adaption via *dltB* polymorphism occurs in companion rabbit isolates and provide further evidence for convergent evolution at this locus across diverse *S*. *aureus* lineages infecting rabbits. In addition to the DltB polymorphisms described previously we have identified six novel amino acid substitutions, Table 1. Using a predicted membrane topology model of the DltB protein, Viana *et al*. noted that the majority of mutations they described were predicted to be in the extracellular loops or proximal to the outer surface of the membrane [17]. Using that model, while none of the novel mutations described here appear to be located extracellularly, four are predicted to be proximal to the outer surface of the membrane (data not shown). Interestingly, the pattern of distinct polymorphisms between the isolates included the two related ST30 isolates, FP01 and FP02, cultured from the same animal. These two isolates shared one amino acid insertion but had a distinct second amino acid substitution. This suggests that the selective pressure exerted on *dltB* by the rabbit host may be acting to drive divergent evolution within clones within the same individual host. Although the independent acquisition of two related strains with divergent *dltB* alleles cannot be excluded.

To conclude, we have used antimicrobial susceptibility testing and whole genome sequencing to characterise *S*. *aureus* isolates from companion rabbit. Isolates came from a diverse bacterial population, including three new STs and two new *spa* types. While antimicrobial resistance was uncommon, except for penicillin resistance, isolates possessed a number of virulence factors consistent with the ability to cause severe abscesses in companion rabbits. The availability of these genome sequences will underpin improved understanding of disease pathogenesis, clinical management and pathogen surveillance in this popular companion animal.
